# An Economic Analysis of Pigeonpea Seed Production Technology and Its Adoption Behavior: Indian Context

**DOI:** 10.1155/2016/7973638

**Published:** 2016-07-13

**Authors:** Govind Pal, Radhika Channanamchery, R. K. Singh, Udaya Bhaskar Kethineni, H. Ram, S. Rajendra Prasad

**Affiliations:** ICAR-Directorate of Seed Research, Mau, Uttar Pradesh 275103, India

## Abstract

The present study was based on primary data collected from 100 farmers in Gulbarga district of Karnataka, India, during the agricultural year 2013-2014. Study shows that average land holding size of pigeonpea seed farmers was higher in comparison to grain farmers and district average. The study illustrates a ratio of 32 : 68 towards fixed and variable costs in pigeonpea certified seed production with a total cost of ₹ 39436 and the gross and net returns were ₹ 73300 and ₹ 33864 per hectare, respectively. The total cost of cultivation, gross return, and net return in pigeonpea seed production were higher by around 23, 32, and 44 percent than grain production, respectively. Hence, production of certified seed has resulted in a win-win situation for the farmers with higher yield and increased returns. The decision of the farmer on adoption of seed production technology was positively influenced by his education, age, land holding, irrigated land, number of crops grown, and extension contacts while family size was influencing negatively. Higher yield and profitability associated with seed production can be effectively popularized among farmers, resulting in increased certified seed production.

## 1. Introduction

Pigeonpea (*Cajanus cajan* (L.) Millsp.) is one of the protein-rich legumes of the semi-arid tropics grown throughout the tropical and subtropical regions of the world. In India its major area is lying between 14° and 28°N latitude, where the majority of the world's pigeonpea is produced [1]. According to FAO statistics [2], worldwide pigeonpea was grown in about 4.23 million hectares with a production and productivity of 4.68 million tons and 751 kg/ha, respectively. India is the largest producer of pigeonpea accounting for 66 percent of total production and the other major pigeonpea producing countries are Myanmar (17.09 percent), Malawi (6.15 percent), Kenya (4.36 percent), and United Republic of Tanzania (5.29 percent). Pigeonpea ranks second after chickpea among all the pulses in the country and normally cultivated during* kharif* season. In India, it occupies an area of 3.81 million hectares with a production and a productivity of 3.07 million tons and 806 kg/ha, respectively [3]. Pigeonpea is an important crop of Karnataka state in India and contributing around 18 percent and 12 percent to total area and production, respectively [3].

As far as importance of seed is concerned, it is the vital input for attaining sustained growth. Quality seed production is a specialized activity that paves way for initial assurance towards realization of higher output. The general farm saved seed cannot be substituted for quality seed, as it generally lacks genetic vigour and has poor germination [4]. A sustained increase in agricultural production and productivity depends on development of new improved varieties and adequate supply of quality seed to the farmers at the right time. It is estimated that the direct contribution of quality seed alone to the total production is about 15–20 percent depending upon the crop and it can be further raised up to 40 percent with effective management of other inputs [5]. Various factors influence costs and returns in pigeonpea seed production, affect its profitability, and account to different impacts on adopters of seed production as well as grain producers, which necessitates for studies regarding production economics of quality seed production and its adoption among farmers.

Following the agricultural technology revolution of the 1970s in India, there is huge improvement in adoption of quality seed production technology among farmers. Realizing the potential of quality seed sector, the Government of India (GOI) initiated various policies and projects towards public and private seed sector development in the country. Indian Council of Agricultural Research (ICAR), which is apex body in India for undertaking and coordinating agricultural research, shoulders major responsibility towards varietal development especially in case of pulses, which comes under high volume, low value category. In case of pulses, promising varieties and hybrids reach farmers through various extension activities and government initiatives aimed at promotion of new varieties and quality seed usage in farming. As adoption of quality seed production targets to meet existing regional demand for new and promising varieties of various crops, there is need towards understanding of factors affecting farmers' decision making on adoption. Studies of Mariano et al. [6] and Feder and Umali [7] suggest that farmer's decision to adopt agricultural technology depends on farm household characteristics such as socio-economic, institutional, and environmental factors. According to Alene et al. [8], individual household level analysis is the main approach to adoption studies, where the factors influencing farmers' behavior are analysed in understanding the reasons behind adoption of an improved agricultural technology under question. The adoption study assumption is that there exists an innovation and the study of adoption decisions evaluates determinants of its adoption. Guei et al. [9] reported that improving skills and knowledge of farmers in aspects such as seed storage, seed quality management, marketing, accounting, and assessing new varieties could enhance uptake, spread of new varieties, and improved practices and will help to keep the small-scale seed production enterprises commercially viable. Diverse studies present a range of factors such as gender, age, education, land holding, livestock holding, and extension visits to explain the adoption of technology in farming.

From the above discussion it is obvious that different factors need to be studied for positive or negative influences, which either contribute or undermine the development process in technology adoption regime in Indian seed sector. Considering these facts, the present study was taken with the objectives to examine the socio-economic condition of pigeonpea seed growers, economics of certified seed production of pigeonpea in comparison to grain production, and constraints in certified seed production of pigeonpea and to analyse the factors that governed the farmers' decision to adopt seed production technologies in selected study area of Karnataka state in India.

## 2. Methodology

Umpteen reviews are available on the use of models to analyse the determinants of new technology adoption in farming. The influence of various socio-economic factors on the willingness of decision makers to adopt new technologies has been investigated by a number of studies [10–13]. In most of the studies on adoption behavior, the dependent variable is constrained to lie between 0 and 1; the model used is exponential functions [14]. Adeogun et al. [15] summarized in one of their studies the use and choice of models from various available models for analysis of determinants of technology adoption decisions. The study stated that, in case of adoption behavior, the dependent variable is constrained to lie between 0 and 1; the models used were exponential functions while univariate and multivariate logit and probit models including their modified forms have been used extensively to study the adoption behavior of farmers and consumers. According to Shakya and Flinn [16], probit model is recommended for functional forms with limited dependent variables that are continuous between 0 and 1; logit models are for discrete dependent variables. Both logit and probit models appear similar and the major difference is that the logit model shows distribution which has slightly fatter tails. Dependent variable used in this study is discrete and dichotomous in nature (mutually exclusive and exhaustive) and thus followed binary logit model that contains one dependent variable with two categorical outcomes (if the farmer belongs to category of adopter or not). This study examined more than one independent variable to predict the outcome probability and thus multivariate binary logit model is used for analysis. The logit model, which is based on cumulative logistic probability functions, is computationally easier to use than other types of models and it also has the advantage to predict the probability of farmers adopting any technology [15].

### 2.1. Logit Model

Multivariate logit model and its forms have been used extensively to study the adoption behavior of farmers based on the general recommendation on its use in predicting dichotomous outcomes associated with farmer decisions on adoption. The multivariate logit model as specified below was estimated using the maximum likelihood method.

The two basic equations of multivariate logit regression model are as follows.

*Equation (1)*. The logit model assumes that the underlying explanatory variables are random variables which predict the probability of seed production technology adoption:

(1) $$\begin{array}{c} \pi \left(\;X\;\right)=\frac{e^{\beta 0+\beta 1X1+\beta 2X2+\cdots +\beta pXp}}{\left(1+e^{\beta 0+\beta 1X1+\beta 2X2+\cdots +\beta pXp}\right)}, \end{array}$$

which gives the probabilities of outcome events given the explanatory variables *X*1, *X*2,…, *Xp*.

*Equation (2)*. According to Menard [17], logit can be specified as

(2) $$\begin{array}{c} \text{logit}\left[\;\pi \left(\;X\;\right)\;\right]=\beta 0+\beta 1X1+\beta 2X2+\cdots +\beta pXp, \end{array}$$

which shows that logistic regression is really just a standard linear regression model, once we transform the dichotomous outcome by the logit transform which transforms the range of *π*(*X*) from 0 to 1 to −*∞* to +*∞*, as usual for linear regression.

### 2.2. Empirical Logit Model Specification

The probability of quality seed production adoption is specified as a function of economic and social factors. It is represented as follows:

(3) ln⁡Pi1−Pi=bo+b1EDNi+b2AGEi+b3LHOLDi+b4FAMSIZEi+b5IRRIGLi+b6CROPNOi+b7EXTCONi+Ui,

where  *P*_*i*_ is the probability that the *i*th farmer is adopter of seed production technology, 1 − *P*_*i*_ is the probability that the *i*th farmer is nonadopter of seed production technology, *b*_*j*_ is logit coefficients (*j* = 0,1, 2,…, 7), and *U*_*i*_ is random disturbances (*i* = 1,2, 3,4,…, 100).

The definitions and measurement of explanatory variables are presented in Table 1.

Several independent variables are used in the analyses which are education status of the farmer, size of land holding, size of irrigated land, size of the family, age of the farm household head, number of extension contacts of the farmer, and number of crops grown in his farm. The choice on the above factors is based on the assumption towards the influential capability of these factors in acting as determinants of technology adoption decision by the farmer. Most of the factors used for analysis base their possibility in such a way that the more favorable or intensive the factor might be the more it is likely to contribute towards adopting the new technology. The above logit model and marginal effect of selected variables on the probability of adoption of seed production technology have been analysed using statistical software SAS 9.3.

### 2.3. Data

The data in which the empirical model is based were drawn from a sample size of hundred farmers (includes fifty seed growers and fifty grain growers of pigeonpea) in Karnataka state using random sampling procedure. Structured questionnaire was used in collecting information from the farmers and primary sample survey was conducted in Gulbarga district of Karnataka. Purposive sampling procedure was used for selecting the study area as the district is having highest area under pigeonpea in Karnataka state which was around 56 percent of total area under pigeonpea during 2009-2010 [18]. Dependent variable is dichotomous in the way that farmers, who were using the technology, were categorized as adopters while those not using were nonadopters. Data on socioeconomic parameters, various inputs used in the grain and seed production of pigeonpea, and their costs and returns were collected for the agricultural year 2013-2014. Tabular analysis was used to compare the different values of farm economy and other aspects of farm business and weighted average was used for average analysis.

## 3. Results and Discussion

### 3.1. Land Holding

The data pertaining to average land holding of sample pigeonpea farmers is given in Table 2. The analysis of data shows that the majority of seed farmers belong to medium category (4–10 ha) followed by large (10 ha and above) and semimedium (2–4 ha) category. The overall average land holding size of pigeonpea seed farmers was 9.46 ha followed by grain farmers (3.71 ha) and district average (2.37 ha).

**Figure 1:**
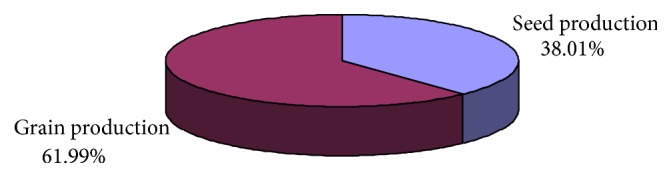
Area under seed and grain production among pigeonpea seed growers.

The area under certified seed production among pigeonpea seed growers is given in Figure 1. Around 38-percent area of pigeonpea seed growers was under pigeonpea seed production and 62-percent area under grain production.

### 3.2. Cropping Pattern

**Figure 2:**
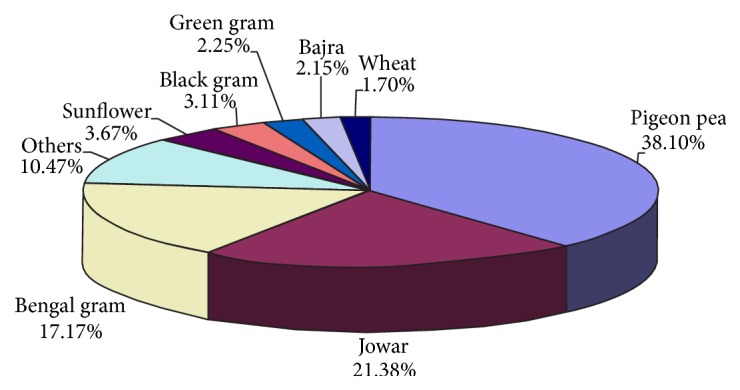
Cropping pattern of study area.

Major crops grown in the study area were pigeonpea, jowar, and Bengal gram. Cropping pattern results of the study area are depicted in Figure 2. This shows that pigeonpea ranked 1st (38.10 percent of gross cropped area) followed by jowar (21.38 percent), Bengal gram (17.17 percent), sunflower (3.67 percent), black gram (3.11 percent), green gram (2.25 percent), bajra (2.15 percent), wheat (1.70 percent), and others (10.47 percent). The cropping intensity of the study area was 109.

### 3.3. Irrigation

**Figure 3:**
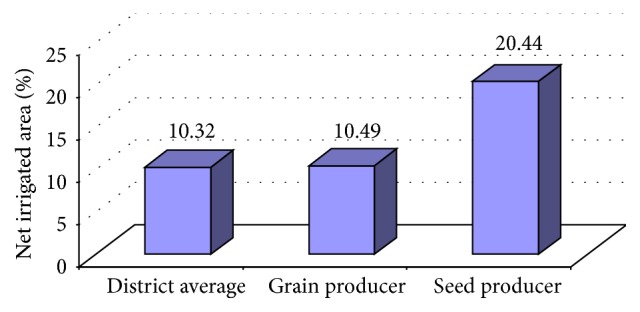
Irrigation pattern in study area.

Irrigated area in the study area is presented in Figure 3. In Gulbarga district, only 10.32-percent area was irrigated, while net irrigated area of pigeonpea grain producer and seed producer was 10.49 and 20.44 percent, respectively. The major source of irrigation was well and tube well (around 66-percent irrigated area).

### 3.4. Pigeonpea Varieties

**Figure 4:**
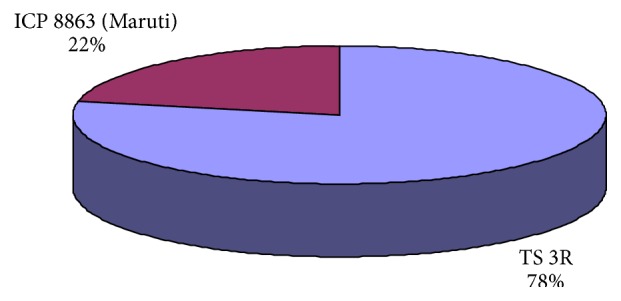
Varieties used for pigeonpea seed production.

Varieties used by seed growers in the study area are presented in Figure 4. Around 78-percent area was under TS 3R and 22-percent area under ICPL 8863. The major characteristics of these two varieties are presented in Table 3.

### 3.5. Economics of Pigeonpea Seed Production

The costs and returns of pigeonpea certified seed production are presented in Table 4. The ratio of fixed and variable cost in pigeonpea seed production was 32 : 68. Human labour was the major component of cost on inputs applied for seed production of pigeonpea; its share in total costs was about 32.46 percent, which was followed by bullock and machine labour accounting for about 12.29 percent of the total cost of pigeonpea seed production. The share of seed cost to total input cost was about 2.49 percent. Cost of manures and fertilizers accounted for about 8 percent and cost of plant protection measures accounted for about 6.85 percent. The total cost in certified seed production of pigeonpea was *₹* 39436 per hectare. The average yield of pigeonpea quality seed and reject seed was 12.5 quintal and 1.7 quintal, respectively, with a byproduct turnout of 31 quintal. The gross and net returns were *₹* 73300 and *₹* 33864 per hectare, respectively.

### 3.6. Comparison of Pigeonpea Grain and Seed Production

**Figure 5:**
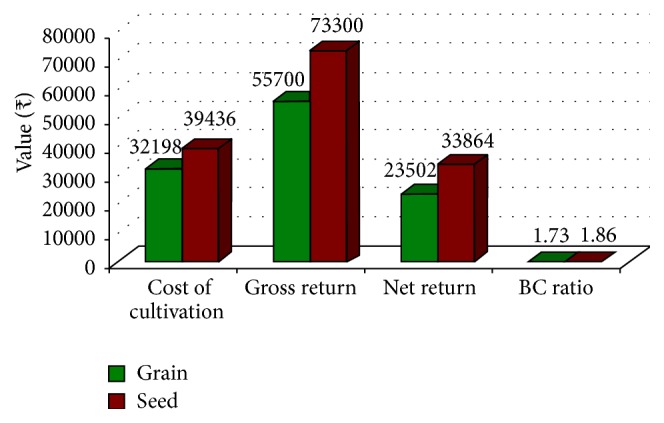
Costs and returns in pigeonpea grain and seed production.

The total cost of cultivation in pigeonpea seed production was around 23 percent higher than grain production, while gross return was about 32 percent higher in seed production (*₹* 73300/ha) than grain production (*₹* 55700/ha). Consequently, net return from seed production of pigeonpea was 44 percent (*₹* 33864/ha) higher than grain production (*₹* 23502/ha). Hence, production of certified seed has resulted in a win-win situation for the farmers with higher yield and increased returns. Graphical presentation of costs and returns in pigeonpea grain and seed production is given in Figure 5.

### 3.7. Constraint Analysis

The factors constraining adoption of pigeonpea seed production technology as perceived by grain producers are presented in Table 5. Small holding size was the most important constraint hindering adoption of pigeonpea seed production technology, as opined by 76 percent of the farmer respondents. The other reasons constraining seed production technology were nonavailability of labour, nonavailability of quality seed, high cost of cultivation, and lack of knowledge and marketing of product, in that order.

### 3.8. Adoption Behavior

The logit framework discussed has described that probability of a respondent to adopt seed production technology was dependent on the socio-economic characteristics of the respondents, that is, education, age of household head, land holding, family size, irrigated land, number of crops grown, and extension contacts. In the present specified logit model, model fit statistics shows that the likelihood ratio chi-square value of 59.0828 with a *p* value of <0.0001 describes that our model as a whole fits significantly. The Score and Wald tests also indicate that the model is statistically significant. The estimates of the logit model are presented in Table 6. Logit model revealed that the decision of the farmer on adoption of seed production technology was positively influenced by his education, age, land holding, irrigated land, number of crops grown, and extension contacts while family size influenced negatively on adoption of seed production technology. Only two variables, out of seven variables included in the model were significant. Extension contacts were significant at 5-percent probability level and land holding was significant at 10-percent probability level. Both significant variables were positively influencing the farmers' decision on adoption of seed production technology. All the other variables included in the model were non-significant.

To analyse the magnitude of variable marginal effect of variables on the probability of adoption has been calculated. Marginal effect of selected variables on the probability of adoption of seed production technology is also presented in Table 6. All variables have positive marginal effect on probability of adoption for seed production technology except family size. Variable extension contact has highest marginal effect on probability of adoption (mean value 0.1038) indicating that each more number of extension contact is likely to have 10.38 percent more adoption of seed production technology by the farmers. Similarly, each more unit of irrigated land is likely to have 5.86 percent more adoption of seed production technology by the farmers.

## 4. Conclusion and Implications

Crops such as pulses, particularly major pulse crop of pigeonpea, have the potential for profitable adoption of quality seed production technology which will further result in improvement of current seed replacement rate and varietal replacement rate scenario in pulses. Thus, identification of various determinants of farmers' choice on technology adoption may help in future strengthening of public sector efforts for popularization of the technology.

The results of tabular analysis indicate that the majority of seed farmers belong to medium, large, and semimedium categories. Major crops grown by farmers are pigeonpea (38.10 percent of gross cropped area) followed by jowar (21.38 percent) and Bengal gram (17.17 percent). Results on net irrigated area under pigeonpea grain and seed production are 10.49 and 20.44 percent, respectively. Results on varieties used by farmers show that varieties such as TS 3R and ICPL 8863 are dominating the seed production scenario in the study area. The net return analysis shows that there is 44 percent higher income from certified seed production of pigeonpea (*₹* 33864/ha) than from its grain production (*₹* 23502/ha). Higher return in seed production is mainly due to increased productivity and better price realization of output. The cost of production results indicate that around 23 percent higher cost incurred in case of seed production of pigeonpea because of high labour requirement, seed certification charges, and higher level of other inputs used in its production. Constraints analysis shows that smaller holding size of farmer is the most important factor, which hinders the technology adoption in pigeonpea.

This study was conducted to understand the farmers' adoption decisions on quality seed production with the assumption that farmers maximize their expected utility; a binary choice model with dichotomous decision to adopt the quality seed production technology was applied. A logit procedure was used for fitting the model. The probability of adoption of quality seed production technology was assumed to be determined by factors such as farm size, education status, age, land holding size, irrigated area, number of crops grown, and contact with extension agents. Individual farmer responses were evaluated after estimation of logit model and marginal effects were computed to understand the effects of changes in the independent variables in the model on adoption probability. The results indicate that education status, age, land holding size, irrigated area, number of crops grown, and extension contact are positively influential in a farmer's decision to adopt quality seed production technology while size of the family shows a negative influence towards adoption decisions. Only two variables, that is, extension contact and size of land holding, are positively significant in the results. Pigeonpea farmers with larger farms are more likely to adopt this technology. Likewise, farmers who have more extension contacts are more likely to adopt, which shows the importance of extension and training programmes towards determining the farmer adoption decisions. Therefore, focused efforts towards training and education by extension personnel for promoting quality seed production technology will increase the probability of adoption of the particular technology among farmers.

This study suggests that higher yield and profitability in seed production may be popularized among the farming community through more extension efforts to increase the gain from certified seed production among farmers in India. Therefore, Major Implication of this study is towards implementation of awareness programmes on adoption of quality seed production. Also, there is a need for strengthening on programs which will focus on creation of timely seed availability for quality seed production. This study reveals that adoption of certified seed production of pigeonpea in farmers' fields is helpful in providing a profitable enterprise for increasing the net farm income.

## Figures and Tables

**Table 1:** Definition of variables in empirical model.

Independent variable	Definition and measurement
EDN	Education level of farmer, measured in years of education completed
AGE	Age of farmer, measured in years
LHOLD	Size of land holding, measured in hectare
FAMSIZE	Family size, measured in number of members in the family
IGRRIGL	Size of irrigated land, measured in hectare
CROPNO	Number of crops grown by farmer in a year, measured in number
EXTCON	Extension contact by farmer, measured in number

**Table 2:** Average land holding of sample pigeonpea farmers.

Land holding particulars	Gulbarga district^*∗*^	Sample pigeonpea farmers		
		Grain farmers	Seed farmers	Overall
Marginal (<1 ha)	0.62 (20.71)	0.53 (16)	— (0)	0.53 (8)
Small (1-2 ha)	1.47 (37.29)	1.46 (20)	1.51 (6)	1.47 (13)
Semimedium (2–4 ha)	2.70 (28.21)	2.64 (28)	2.78 (26)	2.71 (27)
Medium (4–10 ha)	5.75 (11.97)	5.40 (28)	6.56 (38)	6.07 (33)
Large (10 ha and above)	13.54 (1.82)	13.6 (8)	20.53 (30)	19.07 (19)

Average/total	2.37 (100)	3.71 (100)	9.46 (100)	6.59 (100)

Source: http://www.gulbarga.nic.in/dist_at_glance.pdf (^*∗*^as per 2010-11 census).

Note: figures within the parentheses are percentage of farmers belonging to respective group.

**Table 3:** The major characteristics of varieties used in pigeonpea.

Variety	Source	Year of release/notification	Area of adoption zone/state	Average yield (q/ha)	Days to maturity	Remarks
Maruti (ICPL 8863)	ICRISAT, Hyderabad	1986	AP, Karnataka	10–12	115–160	Resistant to wilt
TS-3R	ARS, Gulbarga	2011	Karnataka	11–17	150–160	*Kharif* and late sown cropping, resistant to wilt

**Table 4:** Costs and returns in certified seed production of pigeonpea (₹/ha).

Sl.	Particulars	Amount (₹)	Percent
1	Human labour	12800	32.46
2	Bullock & machine labour	4846	12.29
3	Seed	981	2.49
4	Irrigation	400	1.01
5	Manures & fertilizers	3155	8.00
6	Plant protection chemicals	2703	6.85
7	Seed certification charges	1250	3.17
8	Interest on working capital	801	2.03
9	Total variable cost (₹)	26936	68.30
10	Total fixed cost (₹)	12500	31.70
11	Total cost	39436	100.00
12	Yield		
a	Seed (q)	12.5	
b	Rejected seed (q)	1.7	
c	Byproduct (q)	31.0	
13	Gross return (₹)	73300	
14	Net return (₹)	33864	
15	BC ratio	1.86	

**Table 5:** Factors constraining adoption of pigeonpea seed production technology.

Particulars	Number of farmers	Percentage
Small holding size	38	76
Nonavailability of labour	34	68
Nonavailability of quality seed	32	64
High cost of cultivation	32	64
Lack of awareness/knowledge	29	58
Marketing of product	22	44

**Table 6:** Logit model for farmers' decision on adoption of seed production technology.

Sl.	Variables	*b*_*j*_ (coefficients)	Marginal effect on probability of adoption	
			Mean value	Standard deviation
1	Education	0.087	0.0110	0.0071
2	Age of household head	0.012	0.0014	0.0009
3	Land holding	0.132^*∗*^	0.0168	0.011
4	Family size	−0.060	−0.0076	0.0049
5	Irrigated land	0.461	0.0586	0.0379
6	Number of crop grown	0.150	0.0191	0.0124
7	Extension contact	0.817^*∗∗*^	0.1038	0.0672
	Constant	−4.857		
	Number of observations	100		

Note: *∗∗* and *∗* indicate significance at 5-percent and 10-percent probability level, respectively.
